# Temporary-tattoo for long-term high fidelity biopotential recordings

**DOI:** 10.1038/srep25727

**Published:** 2016-05-12

**Authors:** Lilach Bareket, Lilah Inzelberg, David Rand, Moshe David-Pur, David Rabinovich, Barak Brandes, Yael Hanein

**Affiliations:** 1 School of Electrical Engineering, Tel Aviv University, Tel Aviv 69978, Israel; 2 Tel Aviv University Center for Nanoscience and Nanotechnology, Tel Aviv University, Tel Aviv 69978, Israel; 3 Sagol School of Neuroscience, Tel Aviv University, Tel Aviv 69978, Israel

## Abstract

Electromyography is a non-invasive method widely used to map muscle activation. For decades, it was commonly accepted that dry metallic electrodes establish poor electrode-skin contact, making them impractical for skin electromyography applications. Gelled electrodes are therefore the standard in electromyography with their use confined, almost entirely, to laboratory settings. Here we present novel dry electrodes, exhibiting outstanding electromyography recording along with excellent user comfort. The electrodes were realized using screen-printing of carbon ink on a soft support. The conformity of the electrodes helps establish direct contact with the skin, making the use of a gel superfluous. Plasma polymerized 3,4-ethylenedioxythiophene was used to enhance the impedance of the electrodes. Cyclic voltammetry measurements revealed an increase in electrode capacitance by a factor of up to 100 in wet conditions. Impedance measurements show a reduction factor of 10 in electrode impedance on human skin. The suitability of the electrodes for long-term electromyography recordings from the hand and from the face is demonstrated. The presented electrodes are ideally-suited for many applications, such as brain-machine interfacing, muscle diagnostics, post-injury rehabilitation, and gaming.

Skin electrodes are a common, non-invasive tool used to record electrical activity from the surface of the body (e.g. electroencephalogram (EEG), electromyography (EMG) and electrocardiography (ECG))^1^,^2^,^3^,^4^. Surface EMG (sEMG), in particular, has been suggested for a wide range of applications, such as brain-machine interfacing^5^,^6^, speech detection^7^ and recording emotions^8^. It has been suggested that sEMG can quantify and report elementary emotions exceeding the reliability of self-reporting and image analysis^9^,^10^. By identifying specific muscle activation, facial sEMG can even discriminate between mood states, including Duchenne (genuine) and non-Duchenne (fake) smiles^8^,^11^.

Despite the clear motivation to use sEMG in clinical and daily activities, long-term and multi-site recording remains a challenge, particularly because of poor signal transmission at the electrode-skin interface^12^,^13^. Traditional electrodes require skin preparation (wiping and abrasion) and electrolytic gel application. These prerequisites are time consuming, demand a specialized technician, and are uncomfortable and possibly painful. Furthermore, signal quality gradually degrades over time as the gel dries out. Clearly, dry, stable and easy to apply skin electrodes are highly desired.

Recently, screen-printed electrodes (SPEs) on temporary tattoo film were reported for epidermal pH monitoring^14^, demonstrating its potential for long-term applications. Screen-printing technology allows straightforward patterning of small electrodes with a wide range of inks (e.g. gold, silver, and carbon)^15^. Most importantly, it allows a great flexibility in substrate material (e.g. plastic sheets, cloth). So far, SPEs have been used primarily in the field of chemical sensing^15^,^16^ and bio-sensing^14^,^17^, with limited application in electrophysiological recordings^18^. Thin film based devices^19^,^20^, and organic electrochemical transistors^21^ were also recently proposed for surface sensing applications.

Here we report the development of highly soft carbon SPEs, which can be placed on the skin for a long duration (hours) in a stable manner, with low electrode-skin impedance, even at low geometric areas^22^. The impedance reduction is essential for high signal to noise ratio (SNR), high-resolution recordings. The electrode-skin impedance was improved by a plasma polymerized 3,4-ethylenedioxythiophene (ppEDOT) coating. Plasma polymerization was chosen as a simple, solvent-free, room temperature process^23^,^24^. Specifically, EDOT was selected owing to its biocompatibility and ionic conductivity^25^,^26^. We demonstrate how the plasma polymerized film improves the electrode capacitance, yielding a capacitance comparable with that of state-of-the-art neuronal electrodes. We further show multi-site sEMG recordings from the hand and face and demonstrate force and facial expression sensitivity.

Electrodes were realized following the scheme presented in Fig. 1. The process builds on standard screen-printing, combined with laser cutting to make holes in a double-sided passivation layer, which also serves as a skin-adhesive material (Fig. 1a). The array is placed on the skin and a conformal contact is achieved. This allows stable recordings even for small electrodes (20 mm^2^; Fig. 1b and Supplementary Fig. 1). To reduce the electrode impedance and to improve the electrical recording performances, we implemented a ppEDOT coating.

Figure 1c,d show typical sEMG recordings from the first dorsal interosseous (FDI) muscle (Fig. 1c). Arrows indicate the onset of the signal following a rest position (1), and application of force against the index finger, activating the FDI (2). The signal amplitude is proportional to the force (discussed later). Next, is a flexion of the index finger towards the thumb (isotonic contraction) (3), extension from the thumb (4), and finally pointing up (5) (see also Supplementary Fig. 2). Multi-site recordings allow signal enhancement through an optimal choice of differential electrode pairs (Fig. 1c). Consistently, electrodes located on two sides of the array obtained best results. The electrodes stability was verified by leaving it on the hand for an extended duration and repeating the recording under the same protocol. After three hours, recordings remained stable (Fig. 1d), with a SNR value of 13 (22 dB). No skin irritation was observed for five individuals for up to ten hours (Supplementary Fig. 5).

We characterized the plasma polymerization process, focusing on chemical and impedance spectroscopy analysis. The chemical structure, morphology and physical properties of the film depends on process conditions (power, monomer vapor pressure and deposition time)^27^. For this study, a new process was developed to achieve maximal impedance reduction. We first demonstrate the X-ray photoelectron spectroscopy (XPS) spectra of ppEDOT films (90 W for 10 min) deposited on glass substrates (Fig. 2a). An increase in sulfur and oxygen components from 0.05% to 0.32% and from 53.3% to 56.6% respectively indicates ppEDOT deposition (Fig. 2b). The atomic ratio of sulfur, as well as the concentration of C-S and S-O components, increased with process time and power. ppEDOT coated carbon SPEs were also tested (Fig. 2c), revealing an increase in surface oxygen from 15.2% to 18.6%, indicating the formation of a film incorporating elements from the monomer, while not retaining the monomer chemical structure, was most likely due to fragmentation in the plasma environment^28^. Finally, the morphology of a ppEDOT/carbon SPE was compared to that of a pristine surface using scanning electron microscope (SEM) (Fig. 2d). Coated and uncoated surfaces appeared similar, indicating an ultrathin coating.

For validating the biocompatibility of the coating, comparative *in vitro* cell survival tests were conducted. Primary neurons were cultured on glass coated with ppEDOT, and their survival was compared with that of cells seeded on a poly-D-lysine coating (a cell adhesion promoting protein), and on uncoated surfaces. No significant differences were found in cell survival up to six days (Supplementary Fig. 6).

The electrochemical properties of pristine and ppEDOT SPEs were explored using cyclic voltammetry (CV) and electrochemical impedance spectroscopy (EIS) in a phosphate-buffered saline (PBS). CV data (Fig. 3a) revealed featureless curves for uncoated and coated electrodes as expected for carbon (the small oxidation peak at 0.1 V is typical to carbon ink due to impurities). Current versus scan rate showed linear dependence (Supplementary Fig. 3a) in accordance with a double layer capacitor model. The DC capacitance was calculated and normalized with the electrode area (12 mm^2^) to derive the specific capacitance (Cs). Cs increased by two orders of magnitude from 0.018 to 1.6 ± 0.1 and 3.3 ± 0.2 mFcm^−2^ for coated electrode (12 and 90 W respectively). 1–3 mFcm^−2^ values are comparable with state of the art values of porous titanium nitride and carbon nanotube electrodes^29^ (with 2–10 mFcm^−2^). The impedance and phase data versus frequency of the coated electrodes in PBS is demonstrated in Fig. 3b and in Supplementary Fig. 3b. Electrode impedance appears to stabilize, especially the carbon/ppEDOT, at moderate frequencies to a typical value reflecting the PBS impedance.

The electrode-skin impedance of SPE and commercial electrodes were studied under similar conditions (Fig. 3). The skin was abraded (and wiped) prior to positioning of all electrode types using a designated abrasive paste, as indicated in the Methods section. It is possible that a more aggressive abrasion of the skin may lower the impedance, yet the gentle preparation used here is more suitable for facial and sensitive skin applications. It should be noted that the skin preparation procedure was identical for both pregelled and dry electrodes. At 1 kHz, coated electrodes have on average 10 times lower impedance than that of pristine electrodes, which in turn have about 10 times larger impedance than that of gelled electrodes. Since commercial electrodes have 15 times the area, it appears that their specific impedance is similar to that of dry ppEDOT/carbon electrodes (Supplementary Fig. 3c). The linear correlation between the impedance and the inverse of electrode surface area was validated by testing electrodes at different sizes (Supplementary Fig. 3d).

To demonstrate the performances of the electrodes, we investigated two scenarios: long-term recording from the FDI (Fig. 4a), and from the zygomaticus major (ZM) muscle of the face (Fig. 4c). Immediately after placing the array, stable recordings were systematically obtained in all electrodes. Frequency analysis generated form the discrete Fourier transform (DFT) of signals recorded with carbon/ppEDOT, and pregelled electrodes (Supplementary Fig. 4c,d) illustrate similar frequency spectra, in accordance with the expected spectra for muscle activation.

Typical noise root mean square (RMS) levels were in the range of 83 ± 20, 66 ± 6 and 74 ± 15 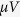 for carbon electrodes with a surface area of 20 mm^2^, ppEDOT/carbon electrode array with a surface area of 20 mm^2^ and commercial pre-gelled electrodes with a surface area of 300 mm^2^, respectively. Noise RMS levels of the differential signals were in the range of 26 ± 11 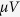 for uncoated electrode array, 25 ± 9 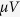 for the ppEDOT electrode array and 22 ± 9 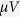 for the commercial. Owing to the high SNR, it was possible to record force sensitive data from the FDI at 0.2–0.5 N with a calibrated spring (Fig. 4b). Interestingly, electrodes without ppEDOT were not sensitive enough to pick the 0.2 N.

Recording from the face was performed to highlight the true strength of the tattoo array. ZM, which pulls the corners of the mouth back and up into a smile, is particularly interesting for emotional expressions and bipolar subjective valence detection^9^. The arrays were placed on the cheek, above the ZM (Fig. 4b). Typical recordings, following different facial expressions (Fig. 4c), are shown in Fig. 4d,e. A clear electrical response is observed while laughing and during a wide smile (Fig. 4d), while a weaker response is measured during a sad expression (Fig. 4e), demonstrating the capacity to differentiate between a smile (observed in 0–7 and 3–4 recordings), and a sad expression (observed only in 0–7 recording). Recordings from the face muscles were conducted on three individuals. The signals presented in Fig. 4d,e are typical results obtained from the same person. Similar differentiation in response to different facial expressions was observed for both individuals.

In this paper novel dry electrodes for long-term sEMG recordings were described. Small (down to 5 mm in diameter) electrodes allow high SNR recording without the need for a gel. This is a divergence from a long held view that asserted otherwise. In fact, many recent efforts to improve dry electrodes focused on skin penetration^30^.

The sEMG system described here suggests many benefits and uses. Foremost, the electrodes are dry, avoiding the complications associated with electrolytic gel, such as skin irritation and gel drying that degrades long-term recordings. The spatial-resolution allows easy electrode placement that simplifies the recording procedure. The exact position of nerves and muscles is no longer a challenge, and can be achieved through different electrode configurations. Finally, the integration with electronic devices and wireless capabilities is straightforward and can be implemented with off-the-shelf electronics.

A key advantage presented here is the use of screen-printing technology combined with a newly developed ppEDOT coating. Screen-printing is cheap and can be readily implemented in large-scale production. Moreover, different materials can be conveniently implemented to support the integration of chemical sensing. Another challenge we addressed is the need for low-temperature and dry surface modification process. ppEDOT was found to enhance the specific capacitance of the SPEs and contributed to a reduction in noise. Better understanding the mechanisms governing the electrode-skin impedance (i.e. carbon layer thickness, adhesive diameter, surface morphology, etc.) will allow further improving the electrodes towards better specificity. The preliminary safety studies presented here reveal that ppEDOT electrodes cause no skin irritation and can be used for hours without discomfort (Supplementary Fig. 5). While focusing on sEMG, other bio-signals, such as EEG, are likely to benefit from the proposed technology. Preliminary investigations reveal similar benefits in placing and recording performances.

To conclude, by demonstrating sEMG recordings from the FDI and the face, we have revealed the potential of these electrodes for future applications. FDI recording is attractive for brain-machine interfacing. As the force applied by the index finger can be readily derived from the sEMG amplitude, sEMG data can be conveniently transformed into a simple communication platform, without the need for a mechanical device. By recording facial expressions, we demonstrated a new route towards recording emotions, opening up new opportunities in digitizing emotions for clinical and social purposes.

## Methods

### Fabrication of screen-printed electrode array

The electrodes were fabricated using a pre-patterned mesh stencil (Sefar Inc.). Printing was accomplished by a manual application of a conductive carbon ink (Conductive compounds) on a blank temporary tattoo paper (Papilio), which served as a substrate. This was followed by curing at 130 °C for 10 min. The electrical conductivity of the carbon film is ~40 Ω/◽. A double-sided adhesive layer (Papilio) was used as a passivation layer. A laser cutter (ELAS Ltd.) was used to define holes for the exposed electrode area. A two-step process was used to prevent overheating of the glue layer: (1) laser intensity of 400 mW and removal of the passivation upper layer, followed by (2) intensity of 800 mW and removal of the remaining two layers. Finally, the adhesive passivation layer with pre-defined holes and the printed electrode were pressed together. To mechanically support contact of the printed electrode array and a ZIF connector (Omnetics), a polyimide tape (Kapton, 3 M) with pre-defined holes was added onto the array bonding pads. The holes in the Kapton film were cut with the same laser cutter with intensity of 400 mW.

### Plasma polymerized EDOT coating

EDOT (Sigma) was plasma polymerized using an RF plasma system (Pico-RF-PC, Diener electronics), operating at a frequency of 13.56 MHz and a monomer vapor pressure of 0.1 mbar. Plasma power of either 12 or 90 W for either 10 or 60 min was used.

### Electrochemical characterization

CV and EIS were performed in PBS (Sigma). CV measurements were conducted using a potentiostat (263A, Princeton Applied Research) under ambient conditions, using a three-electrode cell configuration with an Ag/AgCl reference electrode. DC capacitance was calculated from the slope of the current versus the scan rate according to: 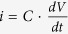, where *i* is the current, *C* is the DC capacitance and *dV/dt* is the scan rate. EIS measurements were conducted under equilibrium conditions by applying 10 mV AC signals using a lock-in amplifier (SR830, Stanford Research Systems) and a potentiostat (263A, Princeton Applied Research). SPE test samples (Dropsens) were used for electrochemical characterization in PBS. Electrode-skin impedance was measured using an amplifier evaluation system (RHD2000, Intan). Pregelled electrodes (Spes Medica) were used for testing (15 by 20 mm) and as reference (35 by 45 mm).

### Surface properties

XPS measurements were performed using a 5600 Multi-Technique System (Physical Electronics). Structural characterization of the printed electrode surface was performed using SEM (JEOL JSM-6700 F).

### Electrophysiology

sEMG recordings were performed using an Intan Technologies amplifier evaluation board (RHD2000). The skin was cleaned (everi, Spesmedica) and dried prior to electrode placement. Force calibration measurements were performed by applying a force against a calibrated spring using the index finger. Noise RMS levels were calculated during the muscle’s relaxation time. SNR was calculated by dividing signal RMS levels (calculated over the period of activation) by noise RMS (2 s window).

### Experiments on Human Subjects

All experiments on human skin were conducted on volunteers in accordance with relevant guidelines and regulations under approval from the Institutional Ethics Committee Review Board at Tel Aviv University. Informed consent was obtained from all subjects.

## Additional Information

**How to cite this article**: Bareket, L. *et al.* Temporary-tattoo for long-term high fidelity biopotential recordings. *Sci. Rep.* **6**, 25727; doi: 10.1038/srep25727 (2016).

## Supplementary Material

Supplementary Information

## Figures and Tables

**Figure 1:**
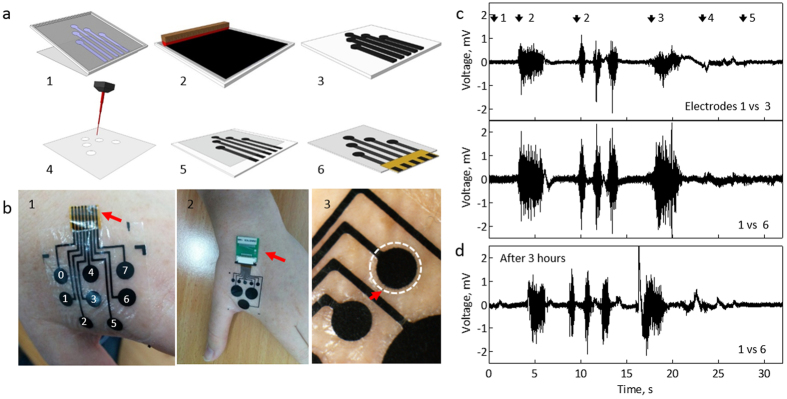
SPEs for long-term sEMG. (**a**) Fabrication scheme: (1, 2) screen-printing onto a temporary tattoo sheet. (3) Electrode array after printing. (4) A double-sided adhesive film is cut with a laser to form the passivation layer. (5) The passivation and the electrode array are aligned and bonded. A plasma step modifies the electrode. (6) A polyimide film with holes is glued to the array to fit into a zero insertion force (ZIF) socket. (**b**) SPE placed on a hand: red arrows highlight (1) the polyimide film; (2) ZIF socket with custom-made printed circuit board (PCB); (3) a hole in the passivation layer. (**c**-top) sEMG recording of the first dorsal interosseous (FDI) using ppEDOT/carbon electrodes (electrode 1 versus 3). Arrows indicate: (1) rest position followed by (2) force application (isometric contractions) on the FDI for 2.5 s and 1 s (repeated three times). (3) Flexion of the index finger towards the thumb, (4) from the thumb and (5) pointing up. (**c**-bottom) electrode 1 versus 6. (**d**) Same as (**c**), three hours after placement.

**Figure 2:**
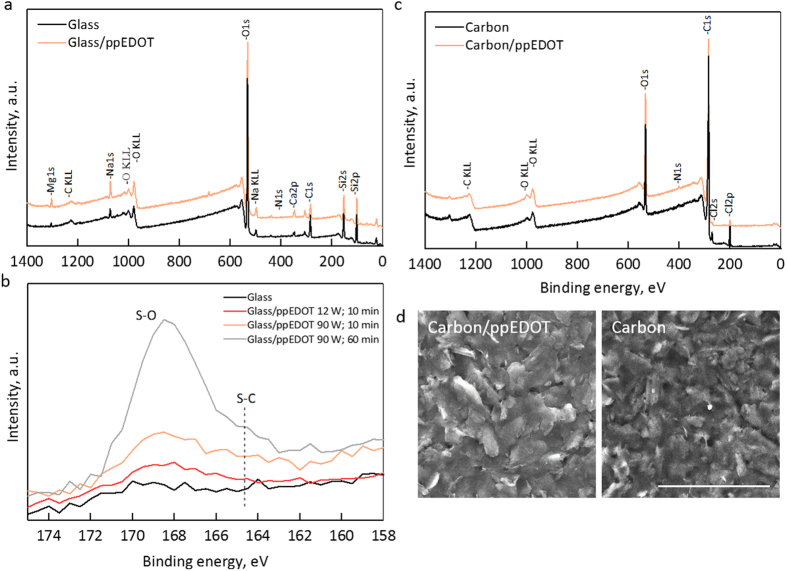
Characterization of ppEDOT films. (**a**) XPS spectra (90 W; 10 min) on glass compared with an uncoated glass. (**b**) Detailed spectra at different plasma deposition power and duration. (**c**) XPS spectra (90 W; 10 min) on a carbon electrode compared with an uncoated electrode surface. (**d**) SEM images of a ppEDOT coated carbon electrode surface (right) compared with an uncoated electrode; scale bar is 50 μm.

**Figure 3:**
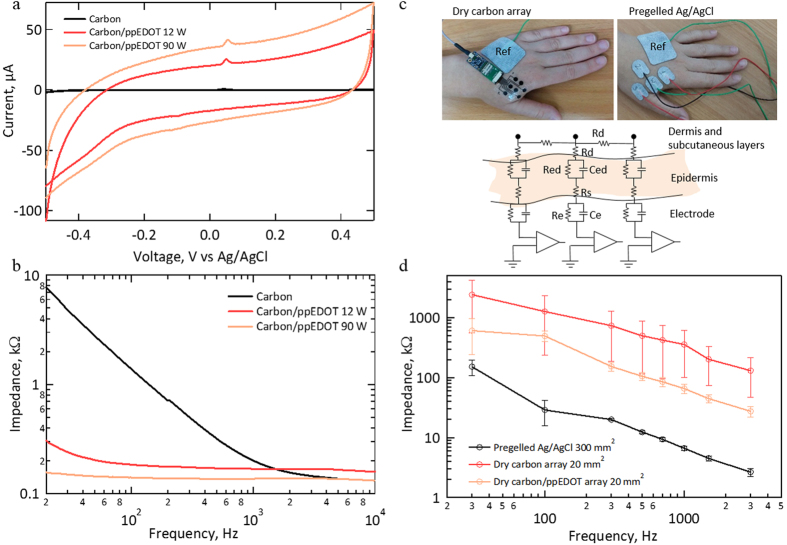
Electrochemical properties of pristine and ppEDOT coated carbon SPE. (**a**) CV scans in PBS (50 mVs^−1^) of ppEDOT electrodes under different deposition power (pristine, 12 and 90 W; 10 min; See also Supplementary Fig. 3a). (**b**) EIS scans in PBS at different deposition powers (pristine, 12 and 90 W; 10 min). (**c**) SPE (left) and commercial electrodes (right) placed on the hand, above the FDI, with a ground electrode placed above the fourth and fifth metacarpals. Inset: an electrode-skin equivalent circuit model. R_e_ and C_e_ represent the resistive and capacitive components of the electrode. R_ed_, C_ed_ and R_d_ denote the RC elements of the epidermis and dermis layers. R_s_ is an effective serial resistance at the interface (i.e. gel or sweat). (**d**) Average electrode-skin impedance versus frequency for commercial (n = 8), carbon (n = 20) and carbon/ppEDOT (90 W for 10 min; n = 13) electrodes with n denoting the number of tested electrodes.

**Figure 4:**
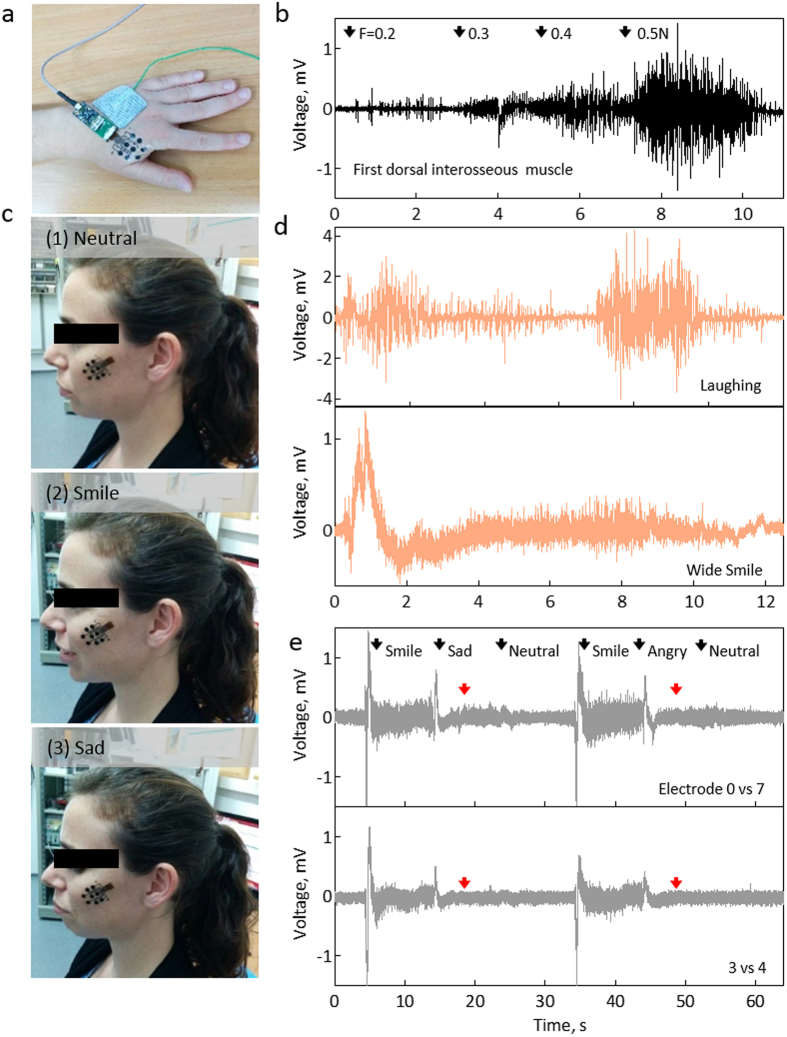
Functional recordings. (**a**) sEMG skin electrodes application for recording FDI activity. (**b**) FDI activity at different forces measured with a calibrated spring. (**c**) Electrode array placed above the ZM of the face during (1) natural, (2) smile and (3) sad expressions. (**d**) Recorded signals distinguishing between a big smile and a laughing expression. (**e**) Differentiation during neutral, smiling, sad and angry facial expressions, using different electrode pairs. The presented signals are typical results obtained from the same person.
