# Corrigendum: Temporary-tattoo for long-term high fidelity biopotential recordings

**DOI:** 10.1038/srep41345

**Published:** 2017-01-23

**Authors:** Lilach Bareket, Lilah Inzelberg, David Rand, Moshe David-Pur, David Rabinovich, Barak Brandes, Yael Hanein

Scientific Reports 6: Article number: 2572710.1038/srep25727; published online: 05 12 2016; updated: 01 23 2017

The authors neglected to cite a previously-published related paper which reports the use of PEDOT:PSS ink-jet printing to realize nanosheets for skin-contact applications. This is listed below as reference 1, and should appear in the introduction as below:

“Thin film based devices^19,20^, and organic electrochemical transistors^21^ were also recently proposed for surface sensing applications”.

should read:

“Thin film based devices^19,20^, organic electrochemical transistors^21^ and PEDOT:PSS ink-jet printed nanosheets^1^, were also recently proposed for surface sensing applications. In the latter example^1^, PEDOT:PSS electrodes printed onto temporary tattoos were used for EMG recordings”.
