# Supplementary material for: Temporary-tattoo for long-term high fidelity biopotential recordings
Source: Sci Rep. 2016 May 12;6:25727. doi: 10.1038/srep25727 (PMC4864418; doi:10.1038/srep25727)
Supplement: Supplementary Information [file srep25727-s1.pdf]

Supplementary Information

Temporary-tattoo for long-term high fidelity biopotential recordings

Lilach Bareket [1,2], Lilah Inzelberg[3], David Rand[1,2], Moshe David-Pur[1,2], David Rabinovich[1], Barak Brandes[1], and Yael Hanein[1,2*]

[1]School of Electrical Engineering, Tel Aviv University, Tel Aviv 69978, Israel

[2]Tel Aviv University Center for Nanoscience and Nanotechnology, Tel Aviv University, Tel Aviv 69978, Israel

[3]Sagol School of Neuroscience, Tel Aviv University, Tel Aviv 69978, Israel

*Corresponding author. Email: yaelha@tauex.tau.ac.il

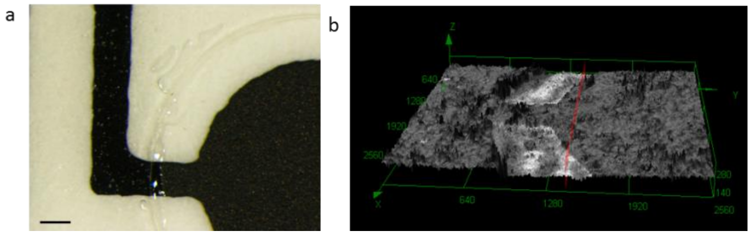

Supplementary Figure 1. Optical microscope image (a), and a three dimensional confocal microscope image (b) of exposed electrode and passivated trace. Scale bar is 500 µm. The hole in the passivation layer allows direct contact with the skin while conducting traces are passivated with a double-sided adhesive layer. The thickness of the carbon and passivation layers is 36±7 and 66±0.7 µm respectively.

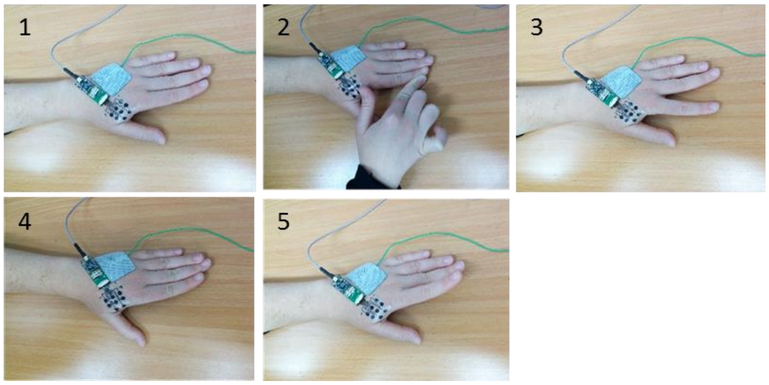

Supplementary Figure 2. Finger movements during recording from the FDI muscle. (1) Rest position followed by (2) application of force (isometric contractions) on the FDI, (3) flexion of the index finger towards the thumb, (4) from the thumb and (5) pointing up.

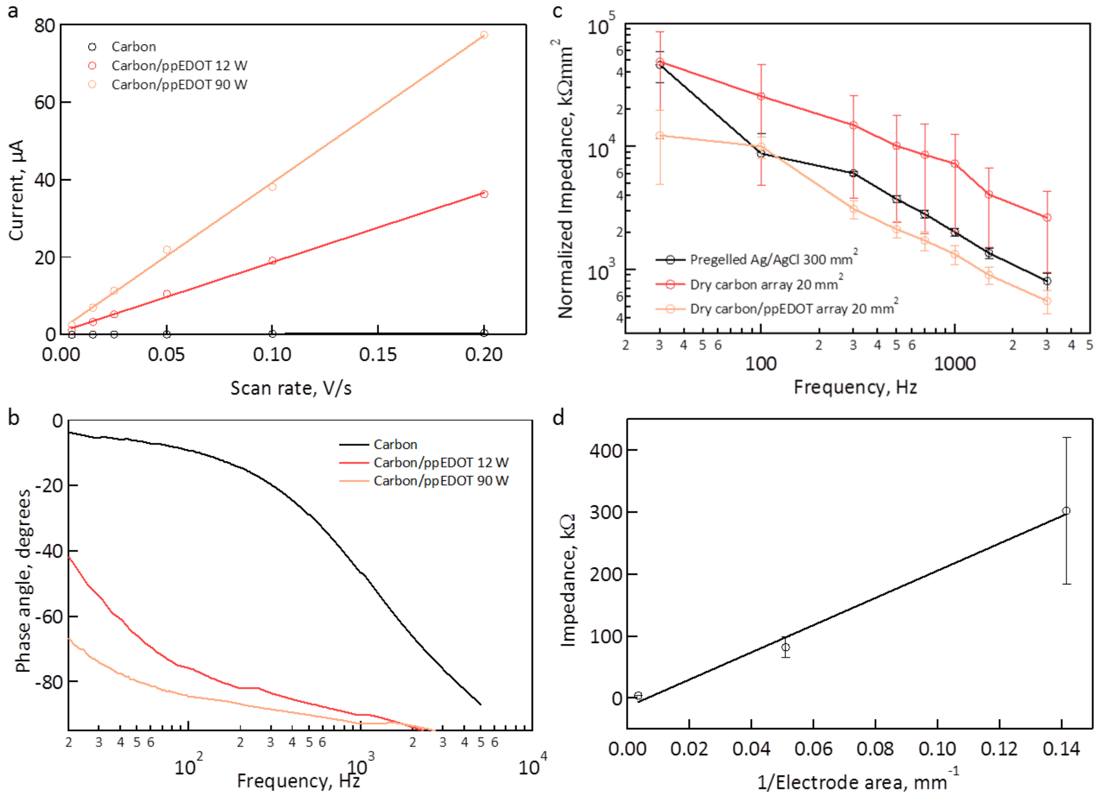

Supplementary Figure 3. (a) Charging current versus scan rate of ppEDOT/carbon SPEs at different plasma deposition powers, solid lines are linear fits (R^2=0.98 for all electrodes). (b) Phase versus frequency. Measurements were performed in PBS with an Ag/AgCl reference electrode and are for a single representative electrode set. (c) Average electrode-skin impedance versus frequency, normalized to electrode surface area. A dry carbon electrode array and commercial Ag/AgCl pre-gelled electrode were tested. Electrodes were placed on the hand, above the FDI, with a large ground electrode placed above the fourth and fifth metacarpals (see also Figure 3c). (d) Average electrode-skin impedance at 1 kHz versus one over electrode surface area, including pregelled Ag/AgCl electrode (15 mm by 20 mm) and Carbon printed electrodes with diameter of 5 mm and 3 mm (inverse of electrode surface area is 0.00033 mm^{-1}, 0.05 mm^{-1} and 0.14 mm^{-1} respectively).

Supplementary Fig. 4 presents the differential voltage versus time for FDI muscle activation using gradual force increase (0.2 N, 0.3 N, 0.4 N and 0.5 N) recorded with carbon/ppEDOT and Ag/AgCl electrodes (Supplementary Fig.4a and 4b respectively). SNR and mean EMG amplitude (calculated at 0.5 N, as the mean RMS Signal amplitude) were 24.22 and 223.53 µV for carbon/ppEDOT electrodes, and

12.79 and 152.68 μV for the pregelled electrodes. Frequency analysis generated form the discrete Fourier transform (DFT) of carbon/ppEDOT, and pregelled electrodes (Supplementary Figure 4c and 4d) illustrate similar frequency spectra, typical to muscle activation.

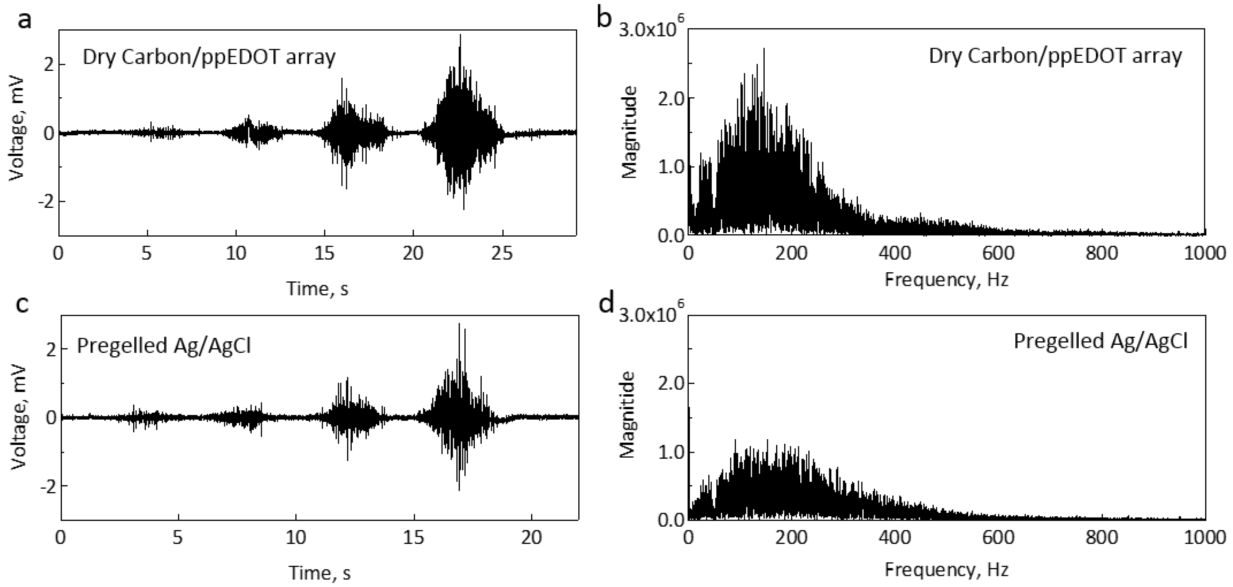

Supplementary Figure 4. Spectral analysis of functional recordings. sEMG recordings of FDI activity during application of gradual forces (0.2 N, 0.3 N, 0.4 N and 0.5 N), measured with a calibrated spring. Each force application was followed by a return to an initial position. (a) Differential voltage versus time for dry carbon/ppEDOT electrode and for Ag/AgCl pregelled electrodes (b). DTF analysis for carbon/ppEDOT electrode (c) and for Ag/AgCl pregelled electrodes (d).

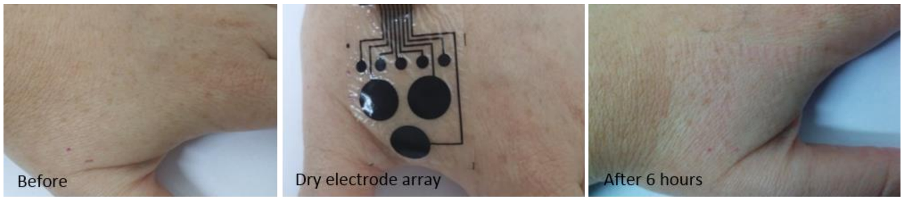

Supplementary Figure 5. Skin irritation tests. Before, during and immediately after application of ppEDOT/carbon dry electrode array for six hours. It appears the skin slightly wrinkles due to application of the sticker and no

irritation was observed. These results were consistent for five individuals, with electrode application times ranging between six to ten hours.

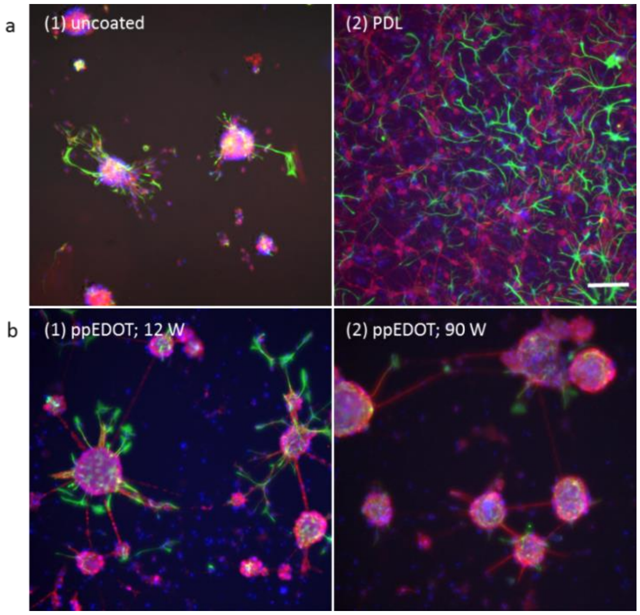

Supplementary Figure 6. In-*vitro* tests. Cultured neural cells on (a) Clean (1) and PDL (a cell adhesion promoting protein) (2) coated glass substrate; (b) ppEDOT coating at low (1) and high (2) deposition power (12 and 90 W respectively; 10 min). Immunostaining after six days *in vitro*: red-neural cells labeled with Neuronal class III beta-tubolin, green-glia cells labeled with anti-glial fibrillary acidic protein, purple-cell nucleus, labeled with DAPI. The ppEDOT coated surfaces support neuronal growth. A clustered morphology appeared on bare glass (a1) and on glass coated with ppEDOT at low and high deposition powers (b1 and b2). Neuronal cells cultured on flat substrates such as glass tend to migrate and rearrange into three-dimensional aggregates due to their poor adhesion to the surface[31], as opposed to cells growing on a substrate coated with a permissive neuronal coating such as PDL[32].

Supplementary methods

In vitro tests

Dissociated cortical cultures were prepared from (E18) Sprague Dawley rat embryos and plated onto substrates at a density of 106 cells/dish (dish diameter was 35 mm). Cultures were maintained at 37 °C with 5 % CO2 and 95% humidity. After six days, in vitro (DIV) cortical cell cultures were fixated using 4% paraformaldehyde (Sigma-

Aldrich). Next, the cells were immunostained using a previously reported protocol[33]. Glia cells and neurons were detected using a mouse anti-glial fibrillary acidic protein monoclonal antibody (GFAP; Chemichon), and neuronal class III beta-tubulin rabbit monoclonal antibody (TUJ1; Covance) respectively. The secondary antibodies included Alexa fluor 488 goat anti-mouse IgG (Molecular Probes) for the detection of GFAP and Alexa fluor 546 goat anti-mouse IgG for detection of TUJ1 (Molecular Probes). Diamidino-2-phenylindole dihydrochloride (DAPI; Sigma Aldrich) was used for nuclei visualization.

Supplementary references

31 Kleinfeld, D., Kahler, K. H. & Hockberger, P. E. Controlled outgrowth of disscociated neurons on patterned substrates. *Journal of Neuroscience* **8**, 4098-4120 (1988).

32 Yavin, E. & Yavin, Z. Attachment and culture of dissociated cells from wmbryo cerebral hemispheres on polylysine-coated surface. *Journal of Cell Biology* **62**, 540-546, doi:10.1083/jcb.62.2.540 (1974).

33 Sorkin, R. *et al.* Process entanglement as a neuronal anchorage mechanism to rough surfaces. *Nanotechnology* **20**, doi:10.1088/0957-4484/20/1/015101 (2009).
